# Metabolic Dysregulation of FC3 Fibrochondrocytes via MDH2 Promotes Intervertebral Disc Degeneration

**DOI:** 10.1111/jcmm.71246

**Published:** 2026-07-02

**Authors:** Xingye Li, Jinbao Tian, Zhongning Xu, Yan An, Jincheng Guo, Da He

**Affiliations:** ^1^ Department of Spine Surgery Beijing Jishuitan Hospital, Capital Medical University Beijing China; ^2^ Beijing University of Chinese Medicine Beijing China; ^3^ Department of Spine Surgery Peking University Fourth School of Clinical Medicine, Beijing Jishuitan Hospital Beijing China

## Abstract

Intervertebral disc degeneration (IDD) is a primary cause of chronic low back pain, yet the specific cell subpopulations and metabolic mechanisms driving its progression remain incompletely understood. We performed an integrative analysis of single‐cell RNA sequencing (scRNA‐seq) and transcriptomic sequencing using public datasets (GSE230809, GSE186542) to characterise cellular heterogeneity in IDD. To elucidate the underlying pathological mechanisms, we employed senescence scoring, transcriptional entropy assessment, pseudotime trajectory inference, and hierarchical weighted gene co‐expression network analysis (hdWGCNA). Metabolic pathway activity was evaluated with scMetabolism, and potential therapeutics were screened using the POINT platform. We identified a key fibrochondrocyte subpopulation, FC3, which exhibits high transcriptional entropy and plays a central role in IDD. The FC3 cluster was further resolved into three functional states: fibrotic, proliferative, and metabolic. Pseudotime trajectory inference indicated that FC3 (proliferative) cells potentially represent a progenitor‐like state, partitioning toward fibrotic and metabolic lineages. Notably, the FC3 (metabolic) state displayed the lowest senescence score and the highest activity in the tricarboxylic acid (TCA) cycle. Through hdWGCNA and cross‐dataset validation, malate dehydrogenase 2 (MDH2) was established as a central hub gene linking TCA cycle activation to the FC3 (metabolic) phenotype. Functional enrichment confirmed MDH2's role in oxidative phosphorylation, fatty acid metabolism, and cellular senescence. Drug screening identified several candidate compounds, including Platycodin D, Irbesartan, and Ergothioneine, whose corresponding targets exhibited specifically enhanced activity within the FC3 (metabolic) subpopulation of degenerated tissues. Our study reveals that metabolic dysregulation in the FC3 fibrochondrocyte subpopulation, driven by aberrant MDH2‐mediated TCA cycle activation, is a critical mechanism promoting IDD. These findings highlight the therapeutic targeting value of the FC3 metabolic state and provide specific candidate compounds for the subsequent development of interventions against IDD.

## 1 Introduction

Low Back Pain (LBP) is a common condition and remains the leading cause of years of disability, and the treatment and management of this condition impose a substantial socioeconomic burden [1, 2]. In the U.S., LBP is reportedly the number one disease among 154 in healthcare spending, with an estimated $100 billion spent [3]. Intervertebral Disc Degeneration (IDD) is closely related to LBP [4, 5]. It involves the progressive deterioration of disc structure and function. It is usually caused by an imbalance between catabolism and anabolism within the Intervertebral Disc (IVD) [6]. The intervertebral disc consists of two main parts: the outer annulus fibrosus and the inner Nucleus Pulposus [7]. The annulus fibrosus is a tough shell of densely packed collagen fibres that maintains the shape of the disc and resists external pressure. The nucleus pulposus, on the other hand, is a water‐rich gel‐like core that is flexible and cushioning, effectively spreading the loads exerted on the spine. In young discs, notochord cells in the Nucleus Pulposus (NP) region promote anabolic processes by up‐regulating the synthesis of extracellular matrix components and down‐regulating the expression of degradative enzymes such as proteases [8]. However, notochordal cells are often lost early in life, which significantly diminishes the disc's ability to maintain normal structure and function and makes it more susceptible to early degeneration [9]. With the loss of notochordal cells, catabolic processes take over, initiating a cascade of events involving the degradation of the extracellular matrix [10]. Although disc degeneration is often viewed as a phenomenon that accompanies the natural aging process, the rate of progression can be exacerbated by a number of risk factors including, but not limited to, genetic background, smoking habits, and obesity status [11]. These factors may further disrupt the dynamic balance within the intervertebral disc by affecting the stability of the microenvironment within the intervertebral disc or by directly acting on cellular metabolic pathways, accelerating the onset and progression of degenerative changes. The prevalence of IDD is gradually increasing, and it has become a major cause of lower back pain, neck pain, and related neurological symptoms in middle‐aged and elderly people.

Recent studies have shown that the onset and progression of IDD is related to a variety of factors, including mechanical loading, trauma, inflammatory responses, oxidative stress, and chronic inflammation, which are considered key factors driving the progression of IDD [12]. For example, a study by Le Maitre and Hoyland revealed that significantly elevated levels of inflammatory mediators such as Interleukin‐1 (IL‐1) and Tumour Necrosis Factor‐alpha (TNF‐alpha) were observed in degenerated and herniated disc tissues as well as the epidural space [13], suggesting that disc degeneration is associated with a significant increase in inflammatory mediators, indicative of an active inflammatory response during disease progression. In addition, further studies have shown that the inherent hypoxic and avascular environment in the nucleus pulposus of the intervertebral disc combined with decreased material recycling capacity and a variety of mechanical stresses and biochemical stimuli combine to create an unfavourable microenvironment. This microenvironment leads to mitochondrial dysfunction and interferes with mitochondrial kinetic homeostasis and quality control mechanisms, which promotes the overproduction of Reactive Oxygen Species (ROS), in addition to the fact that the mitochondria themselves are a major target for ROS attack [14, 15]. The accumulation of ROS causes oxidative damage to mitochondrial DNA, lipids, and proteins, further exacerbating mitochondrial dysfunction. This process creates a vicious cycle or positive feedback loop: initial mitochondrial damage leads to elevated ROS levels, and high levels of ROS in turn exacerbate mitochondrial damage, ultimately leading to a complete imbalance in intracellular homeostasis and irreversible impairment of cellular function.

Currently, the diagnosis of IDD mainly relies on imaging tests, such as X‐ray, CT, MRI, etc., which are used to assess the height and morphology of the intervertebral discs and the presence of abnormalities such as osteophytes [16]. As for the treatment aspect of intervertebral disc degeneration, the treatment strategy mainly includes multi‐dimensional approaches such as conservative treatment, interventional therapy and surgical intervention. Conservative treatment is based on non‐steroidal anti‐inflammatory drugs (NSAIDs) and muscle relaxants for pain relief, combined with physical therapy (e.g., low‐frequency pulsed electrotherapy, core stability training) to improve local microcirculation [16]. In recent years, significant progress has been made in biological therapy, such as percutaneous injection of mesenchymal stem cells (MSCs), which inhibits inflammation and promotes extracellular matrix synthesis through paracrine effects, and platelet‐rich plasma (PRP) growth factors (e.g., TGF‐β, IGF‐1), which can activate the regenerative potential of intervertebral disc cells [17, 18]. For patients with advanced disc degeneration, minimally invasive procedures such as percutaneous endoscopic discectomy (PELD) can effectively decompress and preserve spinal mobility [19], while artificial disc replacement (ADR) can maintain biomechanical function with a titanium‐polyethylene prosthesis [20], and decellularised matrix scaffolds loaded with MSCs in conjunction with dynamic mechanical stimulation can directionally induce fibrous annulus repair [21]. However, all current therapeutic approaches cannot reverse the onset of disc degeneration and can only alleviate the pain of disc patients. In conclusion, disc degeneration is a complex, multifactorial‐driven process involving changes in biomechanics, molecular biology, and other dimensions. A deeper understanding of the pathogenesis of IDD can help develop more effective prevention and treatment methods and improve the quality of life of patients. Future research directions will focus on exploring new therapeutic targets and personalised medicine strategies to address this common and troubling health problem.

## 2 Materials and Methods

### 2.1 Data Sources

The data used in this study were retrieved from GEO (https://www.ncbi.nlm.nih.gov/geo/) and downloaded from GSE230809 and GSE186542 (Table 1). Gene sets for TCA cycle were obtained from the GSEA database (https://www.gsea‐msigdb.org/gsea/index.jsp), and a collection of aging genes was obtained from the article‐A human tissue‐specific transcriptomic analysis reveals a complex relationship between aging, cancer, and cellular senescence [22].

**TABLE 1:** Clinical information of samples from GSE230809.

Sample id	Type	Tissue	Disease state	Age	Sex
GSM7173748	Thompson grade II	Annulus fibrosus	Healthy	21	M
GSM7173749	Thompson grade II	Annulus fibrosus	Healthy	27	M
GSM7173750	Thompson grade II	Annulus fibrosus	Healthy	25	M
GSM7173751	Thompson grade II	Nucleus pulposus	Healthy	21	M
GSM7173752	Thompson grade II	Nucleus pulposus	Healthy	27	M
GSM7173753	Thompson grade II	Nucleus pulposus	Healthy	25	M
GSM7235331	Thompson grade IV	Annulus fibrosus	Diseased	73	M
GSM7235332	Thompson grade IV	Annulus fibrosus	Diseased	56	M
GSM7235333	Thompson grade III	Annulus fibrosus	Diseased	43	M
GSM7235334	Thompson grade III	Annulus fibrosus	Diseased	42	M
GSM7235335	Thompson grade III	Annulus fibrosus	Diseased	63	M
GSM7235336	Thompson grade III	Annulus fibrosus	Diseased	37	M
GSM7235337	Thompson grade IV	Annulus fibrosus	Diseased	68	M
GSM7235338	Thompson grade IV	Annulus fibrosus	Diseased	61	M
GSM7235339	Thompson grade IV	Annulus fibrosus	Diseased	64	M
GSM7235340	Thompson grade IV	Annulus fibrosus	Diseased	63	M
GSM7235341	Thompson grade III	Nucleus pulposus	Diseased	43	M
GSM7235342	Thompson grade III	Nucleus pulposus	Diseased	42	M
GSM7235343	Thompson grade III	Nucleus pulposus	Diseased	63	M
GSM7235344	Thompson grade III	Nucleus pulposus	Diseased	37	M
GSM7235345	Thompson grade IV	Nucleus pulposus	Diseased	68	M
GSM7235346	Thompson grade IV	Nucleus pulposus	Diseased	61	M
GSM7235347	Thompson grade IV	Nucleus pulposus	Diseased	64	M
GSM7235348	Thompson grade IV	Nucleus pulposus	Diseased	63	M

### 2.2 QC, Filtering and Clustering

For independent analyses of single cells and nuclei, individual sample matrices were imported into the Seurat v4.0.0 R package and merged into a unified Seurat object [23]. Cells were filtered based on the following criteria: mitochondrial read percentage < 15%, total RNA counts (nCount_RNA) between 500 and 100,000, and number of detected genes (nFeature_RNA) between 200 and 10,000.

For each dataset, single‐nucleus samples were integrated using the merge function in Seurat. Data normalisation and variance stabilisation were performed using SCTransform, which fits a regularised negative binomial model and regresses out the percentage of mitochondrial reads.

Principal component analysis (PCA) was then conducted, retaining the top 50 principal components (PCs). An elbow plot was generated to determine the optimal number of significant PCs for downstream analyses. Batch effects were corrected using the Harmony() algorithm, leveraging the selected 50 PCs [23]. Uniform Manifold Approximation and Projection (UMAP) dimensionality reduction was subsequently computed based on these batch‐corrected PCs.

Unsupervised clustering was performed using the FindNeighbors and FindClusters functions, again utilizing the same set of significant PCs. Clustering resolution was explored across a range from 0.1 to 1.0, and a final resolution of 0.1 was selected, as it best captured the distinct cell types identified in the single‐nucleus dataset for subsequent analysis. Metadata including experimental conditions and annotated cell type labels were also added to the final Seurat object.

### 2.3 Differentially Expressed Gene (DEG) Analysis and Functional Annotation

First, we employed the FindAllMarkers function to identify unique marker genes for each cell subpopulation, facilitating a better characterisation of their specific functional roles. In this process, the Log2 Fold Change (Log2FC), representing the fold change of gene expression on a log2 scale, served as a key metric for identifying differentially expressed genes (DEGs). The screening criteria for DEGs were set at “|Log2FC| > 0.25” and “*p* value < 0.05” to ensure the inclusion of only those genes with significant expression changes and statistical confidence. Additionally, we used the subset function to extract various combinations of cell populations for a more in‐depth analysis of specific cellular subtypes.

For functional annotation and pathway analysis, we utilised the clusterProfiler package (version 4.6.2) to perform enrichment analysis, which helped identify the unique biological functions associated with these cell subtypes [24]. Furthermore, to quantitatively evaluate the activity of specific gene sets across different cells or conditions, we applied the AddModuleScore function built into the Seurat package. Together, these methodologies enabled a comprehensive characterisation of the relevant biological processes and pathways, providing profound insights into the functional impact and relevance of the identified DEGs within the studied cellular contexts.

### 2.4 Weighted Gene Co‐Expression Network Construction

hdWGCNA is an R package designed for performing weighted gene co‐expression network analysis (WGCNA) on high‐dimensional transcriptomic data, such as scRNA‐seq or spatial transcriptomics. It features a highly modular architecture, enabling the construction of context‐specific co‐expression networks at both cellular and spatial levels [25]. hdWGCNA can identify modules of highly co‐expressed genes and annotate them with biological context through statistical testing and integration of prior biological knowledge. The package operates directly on data formatted as Seurat objects. In this study, we applied the hdWGCNA R package to construct a co‐expression network to identify the gene module most strongly associated with the FC3 cell subpopulation. Specifically, we used hdWGCNA to systematically identify key genes linked to the FC3 cell subtype in intervertebral disc degeneration samples. First, the FC3 cell cluster was isolated from scRNA‐seq data. We then constructed a gene expression correlation matrix, built a weighted gene co‐expression network, and performed module detection. Module‐trait association analysis was conducted to identify gene modules significantly correlated with the FC3 (metabolic) subtype. Finally, intramodular connectivity analysis was used to identify hub genes within the significant modules, aiming to reveal potential functional regulators.

### 2.5 Cell Entropy Assessment

Entropy is a fundamental concept in information theory that measures the uncertainty or randomness of a system. Originally introduced by Norbert Wiener, it was later formalised by Claude Shannon and incorporated into information theory as Shannon entropy, now widely applied in fields such as bioinformatics.

In this study, we use Shannon entropy to assess transcriptional heterogeneity across cells during disease progression and to infer the cell subpopulations that contribute most significantly to the disease state. The calculation proceeds as follows: first, raw gene expression count values are extracted from scRNA‐seq data and normalised using Counts Per Million (CPM). Based on the normalised data, the entropy of each cell is computed using the following formula:

$$H_{i}=-\sum_{i=1}^{n}P\left(x_{i}\right)\log P\left(x_{i}\right)$$

where *P*(*x*_*i*_) represents the probability of gene i's expression level in a given cell, and *n* is the total number of genes. *P*(*x*_*i*_) is calculated as follows:

$$P\left(x_{i}\right)=\frac{{\mathrm{CPM}}_{k}}{\sum_{k=1}^{m}{\mathrm{CPM}}_{k}}$$

where CPM_*k*_ denotes the normalised expression level of gene *k* in the cell, and *m* is the total number of genes.

To further investigate the cellular composition at specific time points, we rank all cells by their entropy values in descending order and select the top 20% highest‐entropy cells, defined as “high‐entropy cells.” A donut plot is then used to visualise the proportion of different cell subtypes within this high‐entropy population, thereby revealing the key contributing subpopulations during critical stages of disease progression.

### 2.6 Pseudotime Analysis

To better elucidate the potential differentiation trajectories within the FC3 cell subpopulation, we first applied CytoTRACE2, a computational tool designed to infer cellular developmental potential and order differentiation trajectories from single‐cell RNA sequencing (scRNA‐seq) data [26]. This method assigns a numerical score to each cell reflecting its differentiation potential: higher scores indicate greater stemness and differentiation capacity, while lower scores suggest a more mature and differentiated state.

In this study, we used the cytotrace2() function in CytoTRACE2 (v1.0.0) to calculate developmental potential scores across cell subtypes, followed by visualisation using the plotData() function to identify putative initiating cells within the FC3 subpopulation. Based on this assessment, we defined the likely root cells for trajectory inference.

Subsequently, single‐cell pseudotime trajectories were constructed using the Monocle2 package in R 4.3.0. A cell dataset was created using the newCellDataSet() function, and analyses were performed using estimateSizeFactors() and estimateDispersions() for normalisation and dispersion estimation. Low‐quality cells were filtered by applying detectGenes() with a minimum expression threshold of min_expr = 0.1, ensuring robustness in downstream trajectory inference [27].

### 2.7 Single‐Cell Metabolic Analysis

To assess metabolic heterogeneity across cell clusters, we employed scMetabolism (v0.2.1), a recently developed computational tool for single‐cell metabolic activity analysis [28]. Built upon the VISION algorithmic framework, scMetabolism quantifies the activity of metabolic pathways in individual cells by integrating standard single‐cell transcriptomic expression matrices. It outputs pathway activity scores for each cell cluster based on databases such as KEGG and Reactome, enabling systematic evaluation of metabolic functional diversity at single‐cell resolution.

In this study, we used the sc.metabolism.Seurat() function with Seurat object input and applied the AUCELL method (Area Under the Cumulative Distribution Function Curve) to calculate enrichment scores for KEGG metabolic pathways across cell subtypes. Finally, heatmaps were generated to visualise the relative activity levels of these metabolic pathways, revealing distinct metabolic profiles—such as those in energy metabolism and biosynthesis—associated with specific cell populations, including the FC3 subpopulation.

### 2.8 Drug Screening Based on POINT

To identify potential therapeutic agents for intervertebral disc degeneration, we performed a network pharmacology‐based drug prediction analysis using the POINT platform (http://point.gene.ac/) [29]. Disease‐associated genes linked to MDH2 were used as input, the “human multilayer network” was selected as the underlying interaction network, and “diffusion‐based feature similarity” was applied to identify candidate drugs with network profiles similar to the disease gene module.

Using this approach, we obtained a ranked list of potential therapeutic compounds. The top 10 drugs and their corresponding target genes were selected as candidate therapeutics. Subsequently, the AddModuleScore() function in Seurat was employed to calculate enrichment scores for each drug‐related gene set across single‐cell populations, assessing their functional relevance within target cell subtypes. Finally, potential therapeutic drugs were prioritised based on a combination of network prediction rankings and module activity scores.

## 3 Results

### 3.1 Single‐Cell Atlas Reveals the Distribution and Functional Heterogeneity of Annulus Fibrosus and Nucleus Pulposus Cells in Intervertebral Disc Degeneration

**FIGURE 1:**
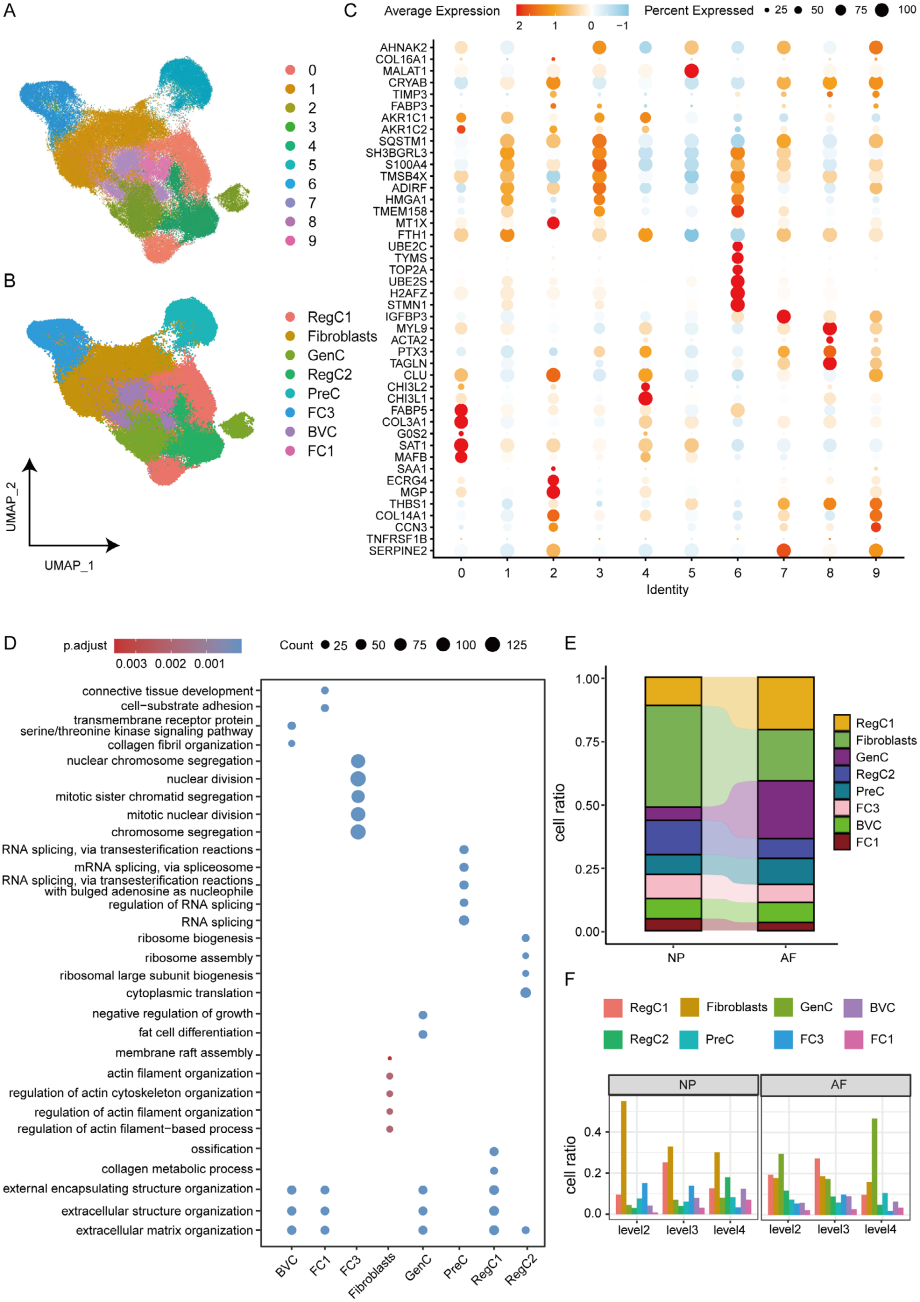
Single‐cell atlas reveals cell states during intervertebral disc degeneration. (A) UMAP dimensionality reduction plot of IDD, showing distinct clustering results. (B) Cell subtype annotation of IDD, with each colour representing a different cell subtype. (C) Dot plot for cell annotation. (D) GO enrichment results for all cell subtypes. (E) Cellular distribution of all cells in the NP (nucleus pulposus) and AF (annulus fibrosus). (F) Proportion of different cell types across disease progression stages in the NP and AF.

In this study, we obtained a single‐cell RNA sequencing dataset (GSE230809) from the GEO database, comprising eight healthy control samples and sixteen intervertebral disc degeneration (IDD) samples. To ensure high data quality, stringent filtering criteria were applied: cells with 200 to 10,000 detected genes (nFeature_RNA), total unique molecular counts between 500 and 100,000 (nCount_RNA), mitochondrial gene content (percent.mt) below 15% were retained. Following quality control, 113,597 high‐quality cells were retained for downstream analysis. Batch effects across samples were corrected using the RunHarmony() function. Principal component analysis (RunPCA) was performed for linear dimensionality reduction, followed by non‐linear visualisation using t‐SNE (RunTSNE) and UMAP (RunUMAP). Clustering analysis identified 10 distinct cell clusters (Figure 1A). Based on canonical marker gene expression, these clusters were annotated as: Regulatory Chondrocyte 1 (RegC1), Fibroblasts, Stable Chondrocyte (GenC), Regulatory Chondrocyte 2 (RegC2), Chondrocyte Precursor (PreC), Fibrocartilage Cell 3 (FC3), Blood Vessel‐like Chondrocyte (BVC), and Fibrocartilage Cell 1 (FC1), among others (Figure 1B,C).

To investigate functional specialisation and compositional differences among cell subtypes during disc degeneration, enrichment analysis and cell proportion profiling were conducted. Functional enrichment revealed distinct biological roles for each cluster. RegC1, RegC2, Fibroblasts, and BVC were significantly enriched in extracellular matrix organisation, collagen metabolism, ossification, and cell‐matrix adhesion pathways, suggesting critical roles in structural maintenance and matrix deposition. In contrast, FC1 and FC3 showed strong enrichment in RNA splicing, ribosome biogenesis, chromosome segregation, and mitotic cell cycle processes, indicating active proliferation and high protein synthesis capacity. PreC exhibited enrichment in translation and post‐transcriptional regulation, consistent with high metabolic activity. GenC was associated with adipocyte differentiation, actin cytoskeleton regulation, and negative regulation of growth, implicating its role in fate determination and morphological stability (Figure 1D).

Single‐cell proportion analysis revealed that the regulatory chondrocyte subpopulation like RegC2 was significantly more prevalent in the nucleus pulposus (NP) than in the annulus fibrosus (AF). Its abundance increased progressively with the grade of degeneration, peaking at level 4, which suggests a potential role in maintaining local homeostasis or modulating inflammatory responses. In contrast, while the overall frequency of chondrogenic progenitor cells (PreC) remained low, a slight upward trend was observed in the AF, potentially hinting at enhanced regenerative capacity at the disc margins. Additionally, the enrichment of FC3 within the NP further confirms the functional heterogeneity of matrix‐producing cells in this region (Figure 1E,F).

### 3.2 Assessment of Senescence Scores and Single‐Cell Entropy Identifies Key Cell Subtypes in Intervertebral Disc Degeneration

**FIGURE 2:**
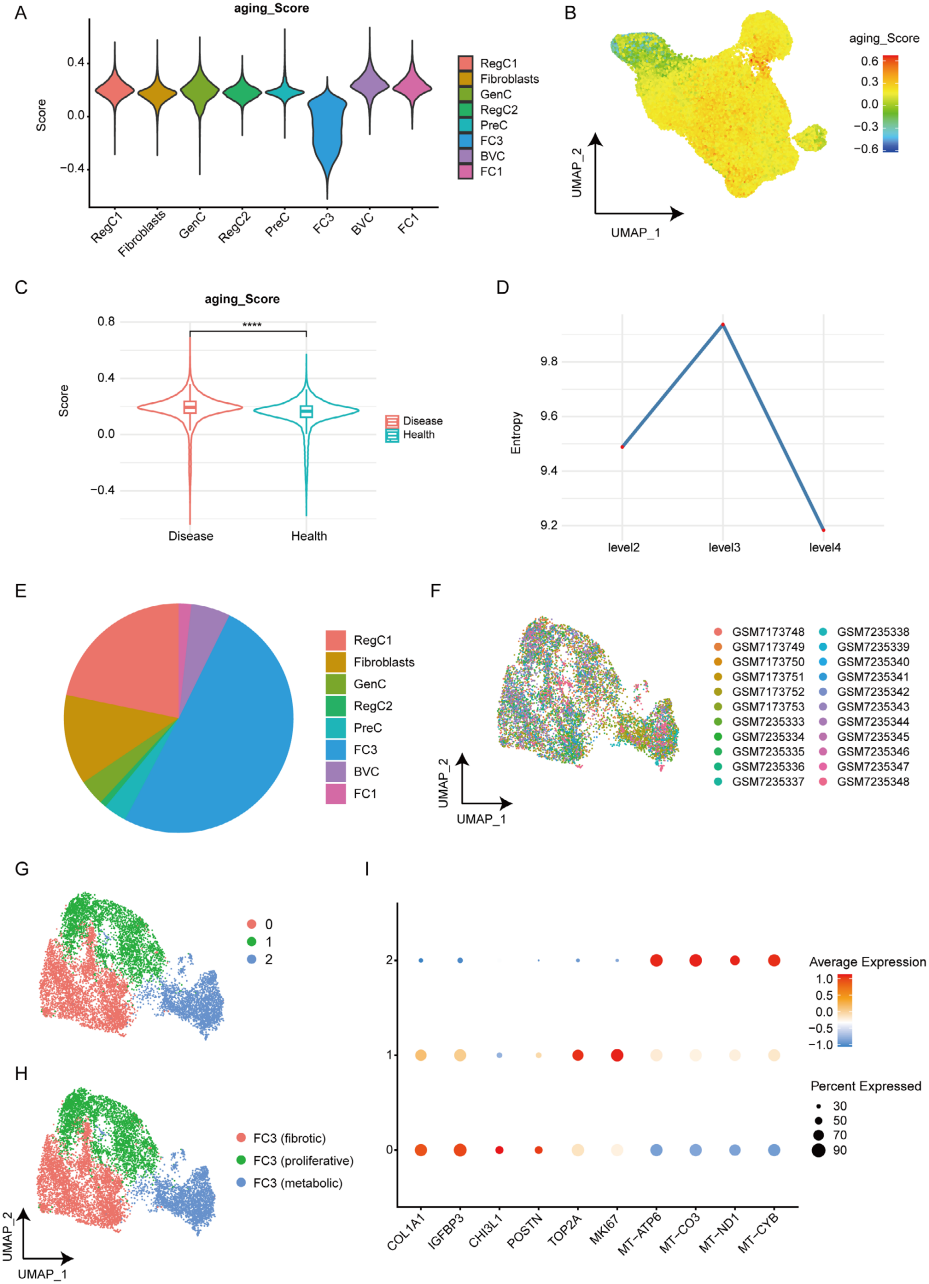
Senescence scores and entropy analysis identify FC3 as a key cell subtype. (A) Senescence scores across all cell populations. (B) FeaturePlot showing the distribution of senescence scores among cells. (C) Comparison of senescence scores between diseased and normal samples. (D) Changes in single‐cell entropy across disease progression stages. (E) Cellular composition of the top 20% of cells with the highest entropy. (F) UMAP plot showing sample distribution of the FC3 population. (G) UMAP dimensionality reduction plot of FC3, displaying distinct clustering results. (H) Annotation of FC3 subclusters, with each colour representing a different cell subtype. (I) Dot plot for FC3 cell annotation.

**FIGURE 3:**
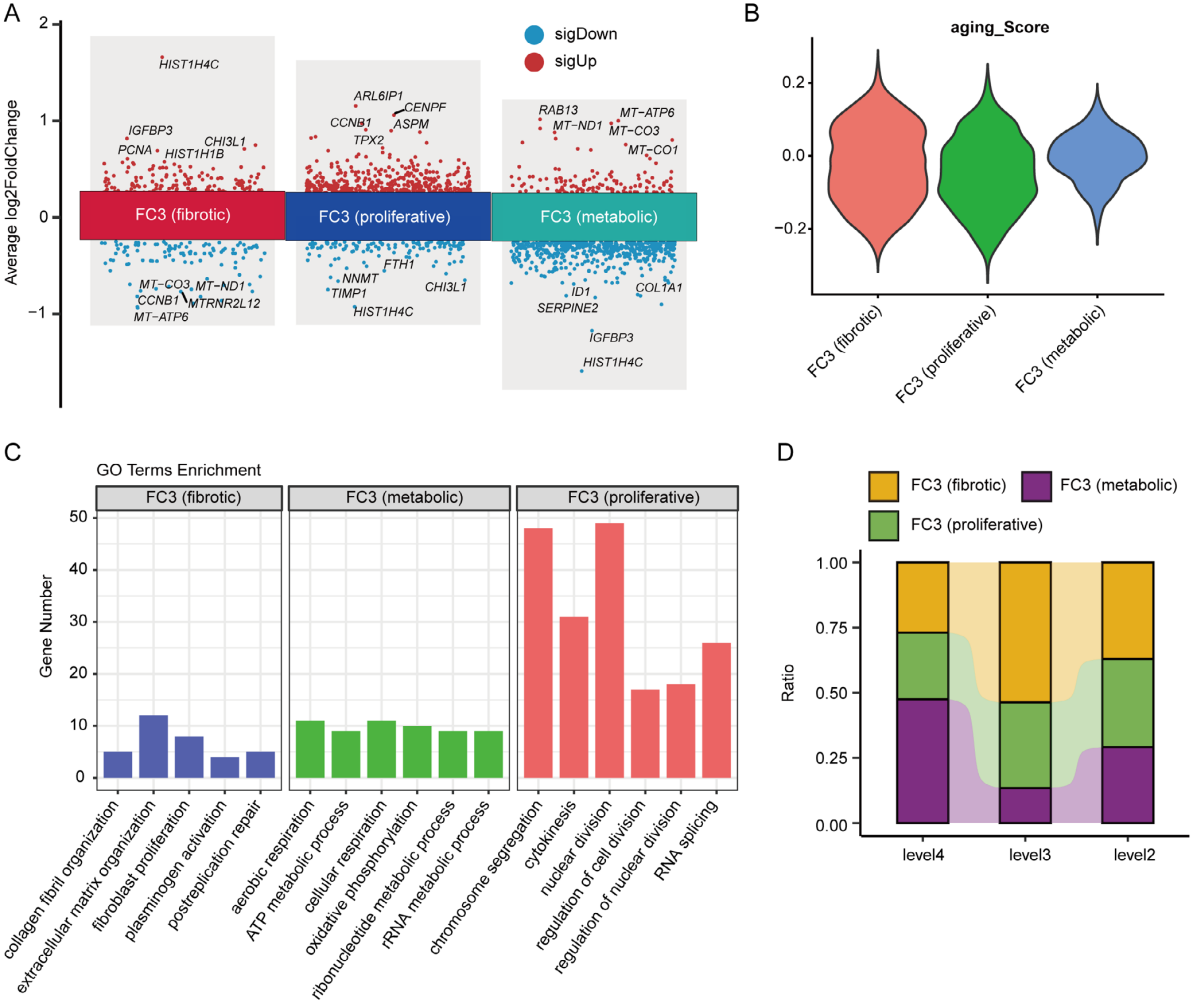
Differential gene expression and enrichment analysis of FC3 cell subtypes. (A) Differentially expressed genes among FC3 cell subtypes. (B) Senescence scores across different FC3 subtypes. (C) Enrichment analysis results for each FC3 cell subtype. (D) Bar plot showing the proportion of FC3 cell subtypes.

Given that intervertebral disc degeneration is often accompanied by aging of the annulus fibrosus or nucleus pulposus, we systematically evaluated the senescence levels of cell subtypes in disease states. Using a senescence‐related gene set defined in the study “A human tissue‐specific transcriptomic analysis reveals a complex relationship between aging, cancer, and cellular senescence,” we calculated senescence scores for each cell population using the AddModuleScore() function in Seurat. The results showed that the FC3 cell subtype had significantly lower senescence scores compared to other cell types (Figure 2A,B), suggesting it may possess enhanced self‐renewal capacity or resistance to aging. However, when comparing degenerated and normal samples, senescence scores were significantly elevated in diseased tissues across all cell types (Figure 2C), consistent with previous findings and indicating that overall tissue aging is a key feature of disc degeneration. To further identify critical transition points in disease progression, we computed transcriptional entropy for each cell. Visualisation revealed a transient peak in entropy at level 3 of degeneration (Figure 2D), suggesting this stage may represent a key window for cell state transition or fate determination. We therefore selected the top 20% of cells with the highest entropy and analysed their composition, finding that FC3 was the most abundant subtype within this high‐entropy population (Figure 2E), implying its dominant role in disease dynamics. Based on this, we extracted the FC3 population for reclustering, which resolved into three distinct subclusters (Figure 2F,G). These were functionally annotated based on marker gene expression: one cluster expressing COL1A1, IGFBP3, and other matrix‐related genes was defined as FC3 (fibrotic); a second cluster enriched for proliferation markers such as TOP2A and MKI67 was designated FC3 (proliferative); and a third cluster specifically expressing mitochondrial genes was annotated as FC3 (metabolic) (Figure 2H,I). Differential gene analysis (FindAllMarkers()) further revealed functional heterogeneity among the three subtypes: FC3 (fibrotic) showed significant upregulation of extracellular matrix‐related genes (e.g., COL1A1, COL2A1), indicating a role in matrix synthesis and maintenance; FC3 (proliferative) was enriched in cell cycle and DNA replication pathways, reflecting high proliferative potential (Figure 3A). Violin plots showed that FC3 (fibrotic) had the highest senescence score, while FC3 (metabolic) had the lowest, suggesting the latter may be in a more primitive or functionally active state (Figure 3B). GO enrichment analysis confirmed these roles: FC3 (fibrotic) was primarily involved in extracellular matrix organisation and collagen formation; FC3 (metabolic) was enriched in glycolysis and fatty acid metabolism pathways; and FC3 (proliferative) was significantly associated with mitosis, chromosome segregation, and DNA replication (Figure 3C). Cell proportion analysis revealed dynamic changes in the composition across level 1 to level 3, with a notable increase in FC3 (proliferative) at level 3 (Figure 3D), suggesting a period of active tissue repair or aberrant proliferation, further supporting the central role of FC3 subpopulations in the progression of intervertebral disc degeneration.

### 3.3 Pseudotime Analysis Reveals Potential Differentiation Trajectory of FC3 Subpopulations

**FIGURE 4:**
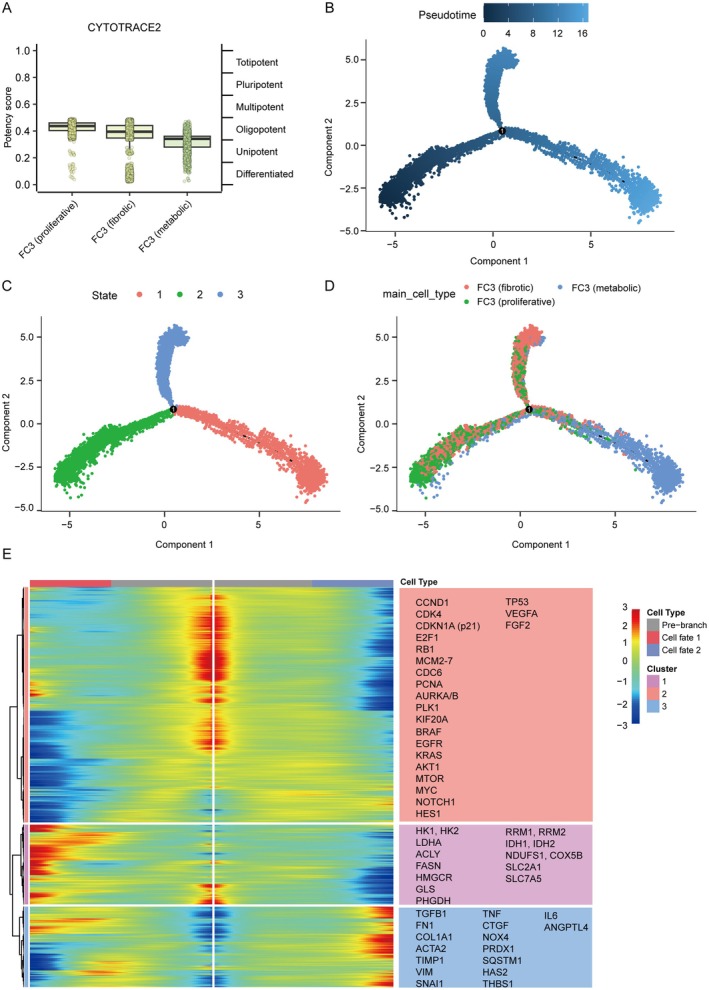
Pseudotime analysis reveals the potential differentiation trajectory of FC3 cell subtypes. (A) Distribution of cell potency scores calculated by the CYTOTRACE2 algorithm. (B) Visualisation of pseudotime trajectory analysis, illustrating a continuous developmental path from early precursor states to terminal differentiated states, revealing dynamic changes during cellular maturation. (C) Cell state partitioning inferred from pseudotime, classifying cells into three major states (State 1–3). (D) Overlay of major cell types (FC3 (proliferative), FC3 (metabolic), and FC3 (fibrotic)) onto the pseudotime trajectory, showing their distribution patterns across the developmental continuum, indicating that multiple cell lineages share or branch from similar differentiation paths. (E) Heatmap of gene expression across different cell states and types. Genes are hierarchically clustered based on expression patterns, clearly revealing multiple transcriptional modules. The colour gradient represents normalised expression levels (red for high expression, blue for low expression), with key genes labelled in the figure.

To further investigate whether the FC3 subpopulations exhibit a potential differentiation trajectory, we first assessed their developmental potential using CytoTRACE2. The results showed that the FC3 (proliferative) subtype had the highest CytoTRACE2 score, significantly exceeding those of the fibrotic and FC3 (metabolic) subtypes, indicating its strongest undifferentiated state and highest differentiation potential (Figure 4A). Based on this, we performed pseudotemporal ordering analysis using the Monocle2 package to reconstruct the differentiation trajectory of FC3 cells. The pseudotime analysis revealed a clear bifurcating path originating from FC3 (proliferative), leading toward either FC3 (fibrotic) or FC3 (metabolic), suggesting that FC3 (proliferative) may serve as a progenitor or transitional state that gives rise to these two functionally specialised terminal subtypes (Figure 4B–D).

To further dissect the dynamic gene expression changes during differentiation, we visualised the expression profiles across cell states using a heatmap, which systematically revealed molecular distinctions between subtypes. The heatmap delineated three major cellular states (State 1, 2, and 3), each characterised by distinct transcriptional patterns and biological functions: State 1 corresponds to FC3 (metabolic), characterised by high expression of genes related to metabolic reprogramming, such as SLC2A1 and FASN, indicating that this portion of cells may be in a metabolic reprogramming stage.; State 2 represents FC3 (proliferative), enriched in cell cycle regulation and DNA replication genes (e.g., MKI67, PCNA), reflecting a transitional state consistent with its role as a differentiation hub; State 3 corresponds to FC3 (fibrotic), which significantly upregulates genes involved in inflammatory response, and mature chondrocyte markers, such as IL6 and COL1A1, indicating terminal differentiation and active participation in extracellular matrix formation and tissue homeostasis (Figure 4E). Together, these findings support a continuous differentiation trajectory within the FC3 population—from a proliferative progenitor state toward functionally specialised fates—providing new insights into cell fate decisions during intervertebral disc degeneration.

### 3.4 Deciphering Key Genes in FC3 Cell Subtypes Using hdWGCNA

**FIGURE 5:**
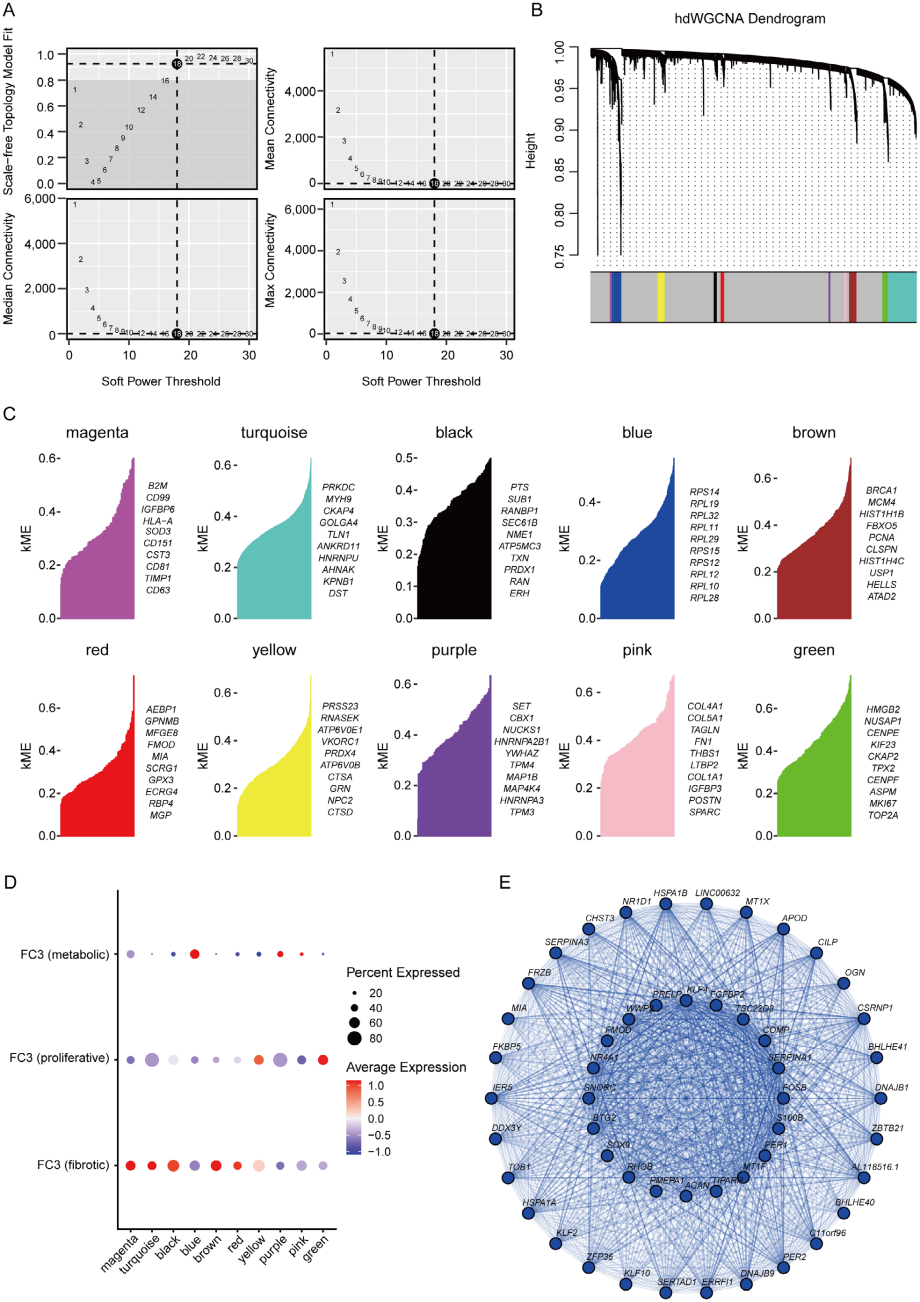
hdWGCNA identifies potential genes associated with cell subtypes. (A) Parameter optimisation for WGCNA network construction. Top left: Trend of the scale‐free topology fit index (*R* ^2^) as a function of the soft power threshold, where *R* ^2^ approaching 1 indicates good scale‐free network properties; top right: Mean connectivity across varying soft thresholds; bottom left: Trend of median connectivity; bottom right: Relationship between mean connectivity and soft threshold. After comprehensive evaluation, a soft power threshold of 18 was selected to ensure a robust network structure. (B) Hierarchical clustering dendrogram generated by the hdWGCNA algorithm, illustrating the clustering relationships of all genes within different co‐expression modules. (C) Association analysis between co‐expression modules and cell types. Each subplot displays the correlation (MAE) between the module eigengene of a given module and three cell types. Colours and curve shapes reflect the expression preference of each module in specific cell types. (D) Expression patterns of modules across different cell types. The colour of each dot represents the average expression level of the module in the corresponding cell type (red for high, purple for low), while the size indicates the percentage of genes expressed (Percent Expressed) within that module. (E) Visualisation of the gene co‐expression network for key modules (e.g., the blue module).

To further dissect the potential regulatory mechanisms underlying each FC3 cell subtype, we constructed and analysed a gene co‐expression network using hdWGCNA, aiming to identify key functional modules and their regulatory relationships. Based on the weighted gene co‐expression network analysis (WGCNA) framework, we determined the optimal soft‐thresholding power (*β* = 18) by integrating the scale‐free topology model and the trend of mean connectivity across different powers, ensuring the resulting network approached a stable scale‐free topology while maintaining sufficient mean connectivity, thus satisfying the topological characteristics of biological networks (Figure 5A). Using this threshold, a hierarchical clustering dendrogram was generated to partition all highly variable genes into multiple co‐expression modules, each represented by a distinct colour (e.g., turquoise, blue, brown), corresponding to groups of genes with coordinated expression patterns that may underlie specific biological functions or regulatory pathways (Figure 5B).

To investigate the functional associations between co‐expression modules and FC3 subtypes, we calculated the correlation between module eigengenes and cell type identities. The analysis revealed that several modules (e.g., magenta, turquoise, black) exhibited significant and distinct correlations with FC3 (metabolic), FC3 (proliferative), and FC3 (fibrotic), suggesting their involvement in subtype‐specific regulatory programs (Figure 5C). To visually depict the expression patterns of each module across the three subtypes, we employed dot plots, where colour intensity reflects module expression levels (red indicating high expression, purple indicating low expression), and dot size represents enrichment significance. The results demonstrated clear cell‐type‐specific preferences: the magenta and brown modules were highly enriched in FC3 (metabolic) and functionally associated with energy metabolism, lipid metabolism, and antioxidant responses, highlighting their central role in maintaining metabolic homeostasis; the turquoise, red, and green modules were predominantly upregulated in FC3 (proliferative) and linked to cell cycle progression and DNA replication; while the black, yellow, and pink modules showed specific high expression in FC3 (fibrotic) and were enriched in extracellular matrix organisation and collagen fibre formation pathways (Figure 5D).

Furthermore, we visualised the top 20 hub genes (highest intramodular connectivity) for each key module using heatmaps to illustrate their co‐expression consistency across different cell states (Figures 5E, S1A–D, and S2A–C). These highly connected hub genes may serve as central regulators within their respective modules, providing critical insights into the functional heterogeneity of FC3 subpopulations and the underlying regulatory networks.

### 3.5 MDH2‐Mediated Metabolic Subtype FC3 and Dysregulation of the TCA Cycle Contribute to Intervertebral Disc Degeneration

**FIGURE 6:**
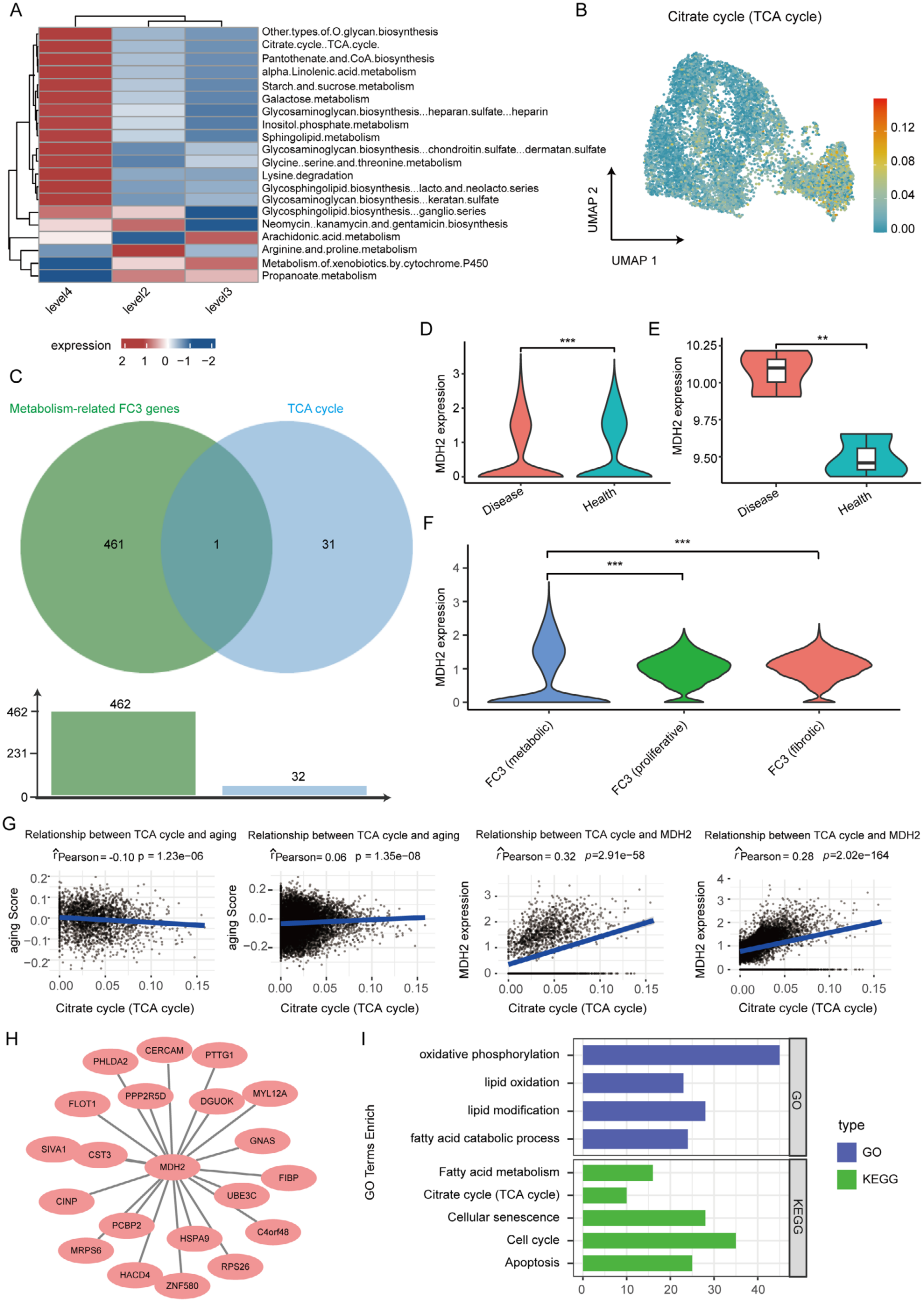
Metabolic analysis identifies MDH2 as a potential key gene. (A) Heatmap showing the expression of metabolic pathways across the three major levels (level 2, level 3, level 4). (B) Feature plot displaying the expression distribution of tricarboxylic acid (TCA) cycle‐related genes at single‐cell resolution. The colour gradient reflects gene expression levels, indicating high TCA cycle activity in specific cell populations and evident metabolic heterogeneity. (C) Venn diagram showing the overlap between TCA cycle‐related genes and FC3‐associated genes linked to metabolic functions. (D) Violin plot comparing the overall expression levels of TCA cycle genes between the degenerative group (disease group) and the healthy group in single‐cell data. (E) A violin plot showing the differential expression of MDH2 (malate dehydrogenase 2) between degenerated tissue and healthy tissue in transcriptome data. (F) Violin plot illustrating MDH2 expression across three functional cell states: FC3 (metabolic), FC3 (proliferative), and FC3 (fibrotic). (G) Scatter plots analysing the correlation between TCA cycle activity and senescence score, MDH2 expression, and cell functional states (From left to right: Correlation between TCA score and senescence score in the FC3 (metabolic) cell subtype; correlation between TCA score and senescence score across all cells; correlation between TCA score and MDH2 expression level in the FC3 (metabolic) cell subtype; and correlation between TCA score and MDH2 expression level across all cells). (H) Gene interaction network centered on MDH2, depicting interactions among its associated genes and highlighting MDH2 as a key metabolic hub (Only the top 20 with the highest correlation are shown). (I) Bar plot showing GO and KEGG pathway enrichment results for MDH2‐associated genes.

Given the presence of a FC3 (metabolic) subtype, we sought to investigate potential differences in metabolic pathways among FC3 subpopulations and across disease progression stages. To this end, we systematically evaluated metabolic activity using the R package scMetabolism. Notably, distinct metabolic signatures were observed across different disease stages: at level 2, enriched pathways were primarily associated with glucose metabolism; level 3 was characterised by inflammation‐related metabolic pathways; and level 4 showed a pronounced shift toward lipid metabolism‐related pathways (Figure 6A). This stage‐specific metabolic reprogramming suggests a dynamic remodelling of energy metabolism during intervertebral disc degeneration.

To further dissect the functional characteristics of the FC3 (metabolic) subtype, we visualised lipid metabolism‐related pathways and found that key energy metabolism pathways, particularly the tricarboxylic acid (TCA) cycle, exhibited significantly higher activity scores in FC3 (metabolic) compared to other FC3 subtypes (Figure 6B), implicating the TCA cycle as a central player in disease progression. Based on this, we retrieved TCA cycle‐related genes from the GSEA database and intersected them with the co‐expression gene set specific to FC3 (metabolic) identified through hdWGCNA. MDH2 (malate dehydrogenase 2) emerged at the core of this intersection, suggesting it may be a key regulatory gene linking the TCA cycle to the functional state of FC3 (metabolic) (Figure 6C).

We then examined the expression pattern of MDH2 in our single‐cell dataset. MDH2 expression was significantly higher in degenerated samples compared to normal tissues. Within the FC3 subpopulation, MDH2 was most highly expressed in the FC3 (metabolic) subtype. To validate this finding, we analysed MDH2 expression in an independent dataset (GSE186542), which consistently showed significant upregulation in diseased tissues (Figure 6D–F), confirming the strong association between MDH2 and disc degeneration.

To further validate the specific role of the TCA cycle in the FC3 (metabolic) subtype, we assessed the correlation between TCA activity and senescence scores, both across all cells and specifically within the FC3 (metabolic) population. While TCA activity showed only a weak correlation with senescence across all cells, a significant negative correlation was observed within FC3 (metabolic), suggesting that high TCA activity may help maintain a lower senescence state. Moreover, the correlation between MDH2 expression and TCA activity was markedly stronger in FC3 (metabolic) than in the overall cell population (Figure 6G), indicating that MDH2's functional role is subtype‐specific and likely central within FC3 (metabolic).

To explore the potential regulatory network of MDH2, we identified genes significantly correlated with MDH2 expression (*p* < 0.05) in the single‐cell data (Figure 6H). GO enrichment analysis revealed that these genes were significantly enriched in “oxidative phosphorylation,” “lipid oxidation,” “lipid modification,” and “fatty acid catabolic process,” highlighting MDH2's crucial role in energy metabolism, particularly in mitochondrial respiration and fatty acid β‐oxidation. KEGG pathway analysis further demonstrated enrichment in “fatty acid metabolism,” “TCA cycle,” “cellular senescence,” “cell cycle,” and “apoptosis,” indicating that MDH2 not only regulates core metabolic processes but may also influence cell fate decisions by modulating energy supply, thereby playing a multifaceted regulatory role in cell proliferation, senescence, and programmed cell death (Figure 6I). These findings collectively underscore the potential significance of MDH2 in the pathogenesis of intervertebral disc degeneration.

### 3.6 Screening for Potential Therapeutic Agents Using the POINT Database

**FIGURE 7:**
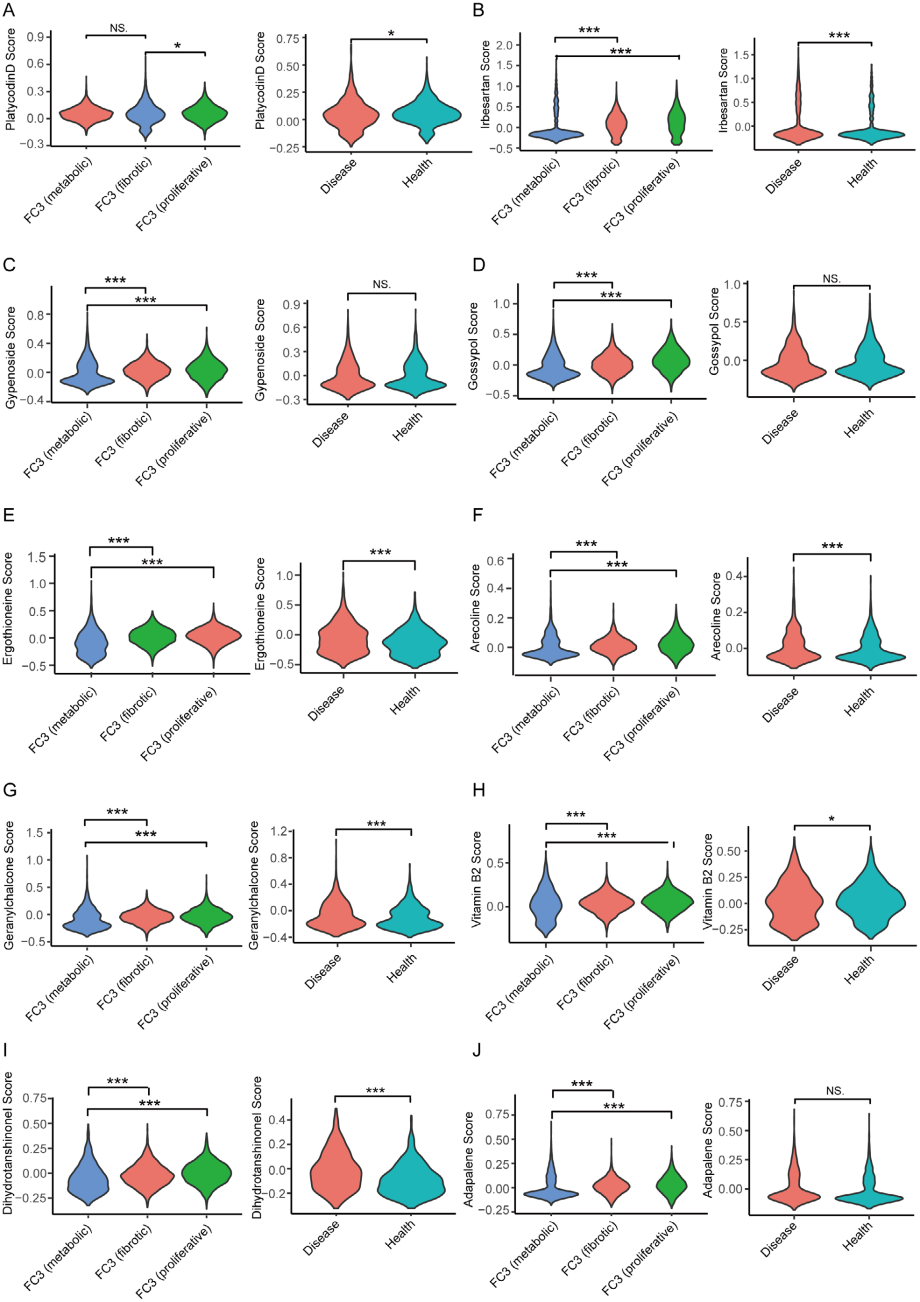
Potential therapeutic drugs based on MDH2 screening. (A–J) Violin plots showing the distribution of module scores for ten drugs—Platycodin D, Irbesartan, Gypenoside, Gossypol, Ergothioneine, Arecoline, Dihydrotanshinone, Adapalene, Geranylchalcone, and Vitamin B2—across three types of cell subpopulations (left) or between disease and healthy samples (right). Red represents the degenerated group (Disease), cyan represents the healthy group (Health), blue represents FC3 (metabolic), orange represents FC3 (fibrotic), and green represents FC3 (proliferative). Statistical significance is marked as follows: *p < 0.05, ***p < 0.001; NS indicates no significant difference.

To explore potential pharmacological intervention strategies for MDH2‐mediated intervertebral disc degeneration (IDD), we submitted the gene set significantly correlated with MDH2 expression (*p* < 0.05) to the POINT database for analysis [29]. Using a diffusion‐based functional similarity algorithm operating on a human multi‐level molecular network, we systematically predicted candidate drugs that may regulate this gene module. Subsequently, the top 10 ranked candidate drugs were selected, and their known or predicted target gene sets were extracted from the POINT database for downstream functional evaluation. To further assess the potential therapeutic impact of these drugs within the disease context, we utilised the AddModuleScore() function in Seurat to calculate module activity scores for each drug's target gene set within the key functional subpopulation—FC3 (metabolic)—and compared these scores between degenerated and normal tissues. The results showed that Gypenoside, Gossypol, and Adapalene exhibited no significant differences in module scores between the two groups, suggesting limited regulatory effects on MDH2‐associated metabolic pathways and potentially insufficient clinical efficacy. Additionally, Vitamin B2 (riboflavin) displayed significantly lower module scores in degenerated tissues compared to normal tissues (Figure 7H). This suggests that its targeted metabolic pathways are markedly suppressed during IDD progression, potentially limiting its efficacy as a standalone intervention in advanced pathological microenvironments. In contrast, potential candidate compounds including Platycodin D, Irbesartan, Ergothioneine, Arecoline, Dihydrotanshinone, and Geranylchalcone showed significantly elevated target module scores in the FC3 (metabolic) cells of degenerated tissues. The high functional enrichment of these drug targets in disease‐associated, metabolically active cells suggests a stronger potential to effectively intervene in MDH2‐driven metabolic reprogramming. It is worth noting that some of these drugs have been shown in studies to resist aging caused by oxidative stress. For example, Platycodin D can resist aging by downregulating aging protein markers such as p53, p21, and p16, and by enhancing mitochondrial quality [30]. Another example is ergothioneine, which can resist aging by scavenging ROS and protecting DNA and proteins from oxidative damage. This potential has been confirmed in various models, such as Drosophila and *C. elegans* [31]. In summary, by integrating network pharmacology prediction with single‐cell resolution functional module scoring, this study identified a set of potential candidate compounds with significantly enhanced target activity in the pathological state. These compounds may exert therapeutic effects by modulating the MDH2‐associated metabolic network, potentially reversing mitochondrial dysfunction and energy metabolic imbalance, thereby offering promising therapeutic strategies for intervertebral disc degeneration. However, these findings warrant further functional validation and pharmacological assessment in future studies.

## 4 Discussion

This study systematically elucidates the distribution characteristics and functional heterogeneity of annulus fibrosus (AF) and nucleus pulposus (NP) cells during intervertebral disc degeneration (IDD), by integrating single‐cell transcriptomics, functional enrichment analysis, pseudotime trajectory inference, gene co‐expression network construction, and drug target prediction. We focus on a crucial fibrochondrocyte‐like cell subpopulation, FC3, and deeply dissect its dynamic evolution, metabolic reprogramming, and potential regulatory mechanisms during IDD progression. Our findings not only expand the understanding of the cellular landscape in IDD but also provide novel molecular insights and candidate therapeutic agents for future targeted interventions.

Single‐cell RNA sequencing (scRNA‐seq) has revolutionised the study of biological mechanisms of disease. The technology has been used to identify and characterise unique cell types and subpopulations, thereby elucidating cellular heterogeneity. scRNA‐seq's true value lies in its ability to detect transcriptional alterations or perturbed pathways within specific cell types under pathological conditions. Currently, single‐cell sequencing (scRNA‐seq) technology has been used to resolve cellular heterogeneity and dynamic transcriptomic changes in the intervertebral disc microenvironment, providing unprecedented resolution to unravel the molecular mechanisms of IDD. For example, Zhang et al. systematically revealed the dynamic changes of chondrocyte subpopulations in the course of IDD by scRNA‐seq technology, and for the first time directly correlated ferroptosis with disc degeneration, which provides an important basis for understanding the molecular mechanism of IDD and developing targeted therapies [32]. The study by Tu et al. revealed the dynamic evolution of the multicellular ecosystem during NP degeneration and suggested that degeneration is the result of synergistic effects of multiple cell types rather than abnormalities of a single cell, which provides a theoretical basis for the development of multi‐targeted combination therapeutic strategies for different stages of IDD [33]. The integrated multi‐omics analysis revealed the synergistic degeneration mechanism of NP and AF and the region‐specific pathological phenotypes in the course of IDD, which emphasised the importance of designing precise therapeutic strategies for different intervertebral disc divisions and provided a new theoretical framework for delaying or reversing IDD [34, 35]. Although a substantial body of evidence has established a strong association between intervertebral disc degeneration (IDD) and cellular senescence, and IDD is widely recognised as a prototypical age‐related degenerative disorder [36, 37], the underlying molecular mechanisms remain incompletely understood at single‐cell resolution. With advancing age, the regenerative capacity of disc cells progressively declines, leading to the accumulation of senescent cells within both the annulus fibrosus and nucleus pulposus tissues. These cells exhibit a constellation of conserved phenotypic hallmarks, including irreversible cell cycle arrest, senescence‐associated secretory phenotype (SASP), metabolic reprogramming, mitochondrial dysfunction, reactive oxygen species (ROS) accumulation, and deposition of macromolecular damage products [37, 38, 39, 40, 41]. Functionally impaired senescent cells not only lose their normal matrix synthesis and repair capabilities but also actively disrupt local tissue homeostasis through paracrine signalling by secreting pro‐inflammatory cytokines, matrix metalloproteinases, and growth factors. This aberrant secretory profile promotes extracellular matrix degradation, suppresses matrix synthesis, and induces secondary senescence or functional dysregulation in neighbouring cells, thereby establishing a positive feedback loop that accelerates the loss of structural integrity and deterioration of biomechanical properties in the intervertebral disc [42, 43]. However, current understanding of cellular heterogeneity in senescence, subtype‐specific senescence trajectories, and associated regulatory networks in IDD remains limited. In particular, systematic investigations into senescence‐associated cell state transitions and their molecular underpinnings at the single‐cell transcriptomic level are still in their infancy, highlighting an urgent need for high‐resolution multi‐omics approaches to elucidate the complex pathobiological basis of this condition.

In single‐cell transcriptomics, transcriptional entropy has been widely established as a core biological indicator for assessing cellular differentiation potency (stemness) and fate uncertainty. In the present study, the high‐entropy characteristic exhibited by the FC3 subpopulation during disease progression carries profound dual biological significance. On one hand, established theories suggest that high entropy is positively correlated with cross‐lineage differentiation potential and developmental plasticity. This implies that FC3 may function as a progenitor‐like population within the intervertebral disc tissue, possessing latent regenerative and repair potential [44, 45]. On the other hand, under the persistent stress of a pathological microenvironment, elevated entropy often reflects a loss of coordination in transcriptional programs and an increased “transcriptional noise,” representing molecular disorder [26, 46]. We observed that the entropy of FC3 cells reached its peak at Level 3 of IDD (intermediate degeneration). This phenomenon likely marks a critical “tipping point” in disease progression: due to prolonged exposure to hypoxia, high acidity, and mechanical stress within the disc, the original homeostatic transcriptional program of FC3 cells is disrupted, plunging them into a chaotic transitional state of “fate uncertainty.” In this state, cells lose precise control over specific functional pathways and become more susceptible to pathological fate shifts induced by pro‐inflammatory or pro‐fibrotic signals. Consequently, the high‐entropy state of FC3 is not only a manifestation of its role as a differentiation hub but also the logical starting point for program dysregulation under the degenerative microenvironment, ultimately driving the disease toward an irreversible stage.

This study systematically characterised the distribution patterns and functional heterogeneity of annulus fibrosus (AF) and nucleus pulposus (NP) cells in intervertebral disc degeneration (IDD) using single‐cell RNA sequencing data (GSE230809). The results demonstrated that the FC3 subpopulation plays a pivotal role in IDD progression. This subpopulation can be further subdivided into three functional states: FC3 (fibrotic) (characterised by high matrix gene expression), FC3 (proliferative) (characterised by high proliferative activity), and FC3 (metabolic) (characterised by high metabolic activity). Notably, FC3 (metabolic) exhibited lower senescence levels. Pseudotime analysis suggested that FC3 (proliferative) may serve as a progenitor state, differentiating into either FC3 (fibrotic) or FC3 (metabolic) lineages. Integrating hdWGCNA and metabolic pathway analysis, we found that the TCA cycle is significantly activated in FC3 (metabolic), with MDH2 emerging as a central regulatory gene that is markedly upregulated in degenerated tissues. Finally, drug screening using the POINT database identified Platycodin D, Irbesartan, and Ergothioneine as potential therapeutic agents that target the MDH2‐associated metabolic network, showing enhanced module activity specifically in FC3 (metabolic). These findings suggest that these compounds may ameliorate mitochondrial dysfunction and metabolic imbalance in IDD. This study provides a single‐cell resolution view of cellular heterogeneity, dynamic trajectories, and key molecular mechanisms in IDD, offering novel therapeutic targets and candidate drugs for precision intervention.

The core pathological features of intervertebral disc degeneration (IDD) encompass not only progressive extracellular matrix degradation and cellular senescence/apoptosis, but also a severe imbalance in the local metabolic microenvironment. Under physiological conditions, the intervertebral disc—particularly the avascular nucleus pulposus (NP)—relies on limited glucose supply and primarily maintains energy metabolism and cellular homeostasis through glycolysis. However, during degeneration, nutrient supply becomes further restricted, leading to a harsh microenvironment characterised by hypoxia, low pH, and low glucose levels, which forces cells to undergo metabolic reprogramming. Studies have shown that glycolytic activity is abnormally enhanced in degenerated discs, while mitochondrial function is impaired and oxidative phosphorylation capacity is reduced, resulting in insufficient energy production and lactate accumulation, thereby exacerbating acidosis and inflammatory responses [14, 47, 48]. Furthermore, dysregulated lipid metabolism, amino acid metabolic disturbances, and excessive accumulation of reactive oxygen species (ROS) collectively disrupt the balance between matrix synthesis and degradation, upregulate matrix‐degrading enzymes, and induce cellular senescence and pyroptosis [49, 50]. Therefore, metabolic imbalance is not only a key driver of IDD progression but also provides a novel perspective for understanding its pathogenesis and developing targeted therapeutic strategies.

In this study, the FC3 (metabolic) subpopulation exhibits distinct biological features, characterised by exceptionally low senescence scores and significantly elevated tricarboxylic acid (TCA) cycle activity. Through hdWGCNA co‐expression network analysis, we identified malate dehydrogenase 2 (MDH2) as the core regulatory gene linking TCA cycle activity to the functional state of this subpopulation. The marked upregulation of MDH2 in degenerated tissues, validated across independent datasets, provides robust evidence for its conserved role in intervertebral disc degeneration (IDD). Functional enrichment analysis revealed that MDH2‐associated genes are involved not only in oxidative phosphorylation and fatty acid metabolism but also deeply in cell cycle regulation, senescence, and apoptosis, suggesting that MDH2 may dictate cell fate by modulating energy metabolism.

Notably, TCA cycle activity in FC3 (metabolic) is significantly negatively correlated with senescence scores, which is highly consistent with the emerging concept that “metabolic reprogramming can delay cellular senescence,” suggesting that this subpopulation may represent a functionally active state with self‐renewal potential.

Malate dehydrogenase 2 (MDH2) is a critical metabolic enzyme localised in the mitochondrial matrix, belonging to the malate dehydrogenase (MDH) family. As a core component of the tricarboxylic acid (TCA) cycle, MDH2 catalyses the reversible oxidation of malate to oxaloacetate while reducing NAD^+^ to NADH [51]. Dysregulation of MDH2 disrupts metabolic and redox balance, leading to excessive ROS accumulation, mitochondrial dysfunction, and cellular stress responses. These disturbances have been implicated in diverse pathological conditions, such as cancer, neurodegenerative diseases, and metabolic syndrome [52, 53, 54, 55]. Intervertebral disc degeneration (IDD) is a complex degenerative disorder closely associated with metabolic dysregulation, oxidative stress, and chronic inflammation [47, 56, 57]. Nucleus pulposus (NP) cells, the primary cellular components of the intervertebral disc, predominantly rely on glycolysis for energy supply. However, the TCA cycle and mitochondrial integrity remain crucial for maintaining cellular homeostasis [58, 59]. Given its central role in metabolic regulation, MDH2 dysfunction may critically influence NP cell survival and disc integrity. We hypothesise that pathological upregulation or aberrant expression of MDH2 could disrupt metabolic homeostasis, lead to excessive ROS generation, and exacerbate mitochondrial dysfunction in NP cells. These alterations can accelerate NP cell apoptosis, degrade the extracellular matrix (ECM), and activate pro‐inflammatory signalling pathways (e.g., NF‐κB), creating a self‐perpetuating cycle that drives IDD progression. Therefore, MDH2 emerges as a potential metabolic hub linking mitochondrial dysfunction to IDD pathogenesis. Investigating its expression, activity, and regulatory mechanisms in degenerative disc tissues may unveil novel therapeutic strategies for mitigating or reversing IDD. Targeting MDH2‐mediated metabolic pathways could offer a promising approach to restore cellular homeostasis and delay disc degeneration.

This finding, coupled with anatomical site analysis, carries profound pathological implications: although the internal environment of the nucleus pulposus (NP) is physiologically hypoxic and typically relies on glycolysis to maintain homeostasis, our analysis shows that the NP‐enriched FC3 (metabolic) subpopulation exhibits abnormally active MDH2‐driven TCA cycle activity. This phenomenon reflects a stress‐induced metabolic reprogramming in cells under degenerative pressure, aimed at compensating for the energy crisis during degeneration by enhancing mitochondrial oxidative phosphorylation. However, the forced activation of the TCA cycle under a persistently hypoxic microenvironment inevitably leads to severe pathological consequences; such an aberrant metabolic switch is highly prone to inducing the overproduction of reactive oxygen species (ROS), thereby exacerbating mitochondrial damage, oxidative injury, and cellular stress. This may provide a potential molecular explanation for why the abnormal upregulation of MDH2 is significantly positively correlated with the severity of IDD. Therefore, given the high abundance of FC3 in the NP and its pathological metabolic characteristics, targeted intervention against MDH2 to correct metabolic dysregulation within the nucleus pulposus holds significant clinical translational value.

Admittedly, this study has several limitations. First, data sources and sample heterogeneity: All data in this study were derived from public databases. Therefore, inherent heterogeneity may exist across different datasets regarding sample origins, sequencing technologies, and clinical backgrounds. Second, the inherent contradiction between static snapshots and dynamic trajectories: Single‐cell sequencing data are essentially static snapshots of tissue states, which imposes intrinsic limitations on using computational tools to infer dynamic developmental trajectories. Although Monocle2 simulations suggested a trend of FC3 cells evolving from a proliferative state toward metabolic or fibrotic phenotypes, this process in the actual in vivo environment is inevitably subject to robust feedback regulation by the degenerative microenvironment (e.g., high acidity, hypoxia, and high osmotic pressure). Consequently, actual differentiation trajectories may exhibit bidirectionality or remain locked in specific states; the precision of these dynamic evolutions requires further confirmation through experiments such as lineage tracing. Third, the lack of direct experimental validation: Current conclusions identifying MDH2 and its mediated TCA cycle as key drivers of FC3 cell degeneration are primarily based on bioinformatic predictions and consensus analysis of multicenter data. The precise molecular mechanisms and causal relationships of MDH2 in regulating FC3 cell fate transitions urgently require further elucidation through future in vitro functional assays (such as siRNA knockdown or overexpression) and relevant animal models. Finally, challenges in the clinical translation of candidate drugs: Although candidate drugs identified in this study (e.g., Platycodin D) demonstrated potential in network pharmacology predictions, their penetration capacity, effective concentration, and long‐term safety within the unique avascular anatomical structure and high‐density extracellular matrix of the intervertebral disc still necessitate systematic assessment at both cellular and animal levels.

In summary, this study systematically characterises, for the first time at single‐cell resolution, the cellular heterogeneity of the annulus fibrosus (AF) and nucleus pulposus (NP) in intervertebral disc degeneration (IDD), revealing the central role of the FC3 subpopulation and its metabolism‐driven mechanisms, particularly the aberrant activation of the MDH2‐TCA axis. We propose that FC3 (metabolic) may represent a cell population with functional plasticity that is “misprogrammed” during disease progression, and that targeting its metabolic pathways could potentially reprogram cell fate, thereby slowing down or even reversing disc degeneration. Future research should focus on validating the functional role of MDH2 and exploring novel therapeutic strategies based on metabolic intervention, offering groundbreaking insights into addressing IDD—a major clinical challenge.

## Author Contributions

**Jinbao Tian:** writing – original draft, software. **Da He:** conceptualization, writing – review and editing. **Zhongning Xu:** supervision. **Yan An:** methodology, writing – original draft. **Xingye Li:** conceptualization, writing – original draft. **Jincheng Guo:** writing – review and editing, project administration, resources, data curation.

## Funding

This study was supported by Beijing Nova Program (H020821500190, Z20230484422, 20240484661).

## Ethics Statement

This study is based on secondary analysis of publicly available data from databases such as GEO. It does not involve any new human or animal experiments, nor does it directly access personally identifiable information; therefore, ethical approval and informed consent were not required.

## Consent

All authors have reviewed and approved the final version of the manuscript and authorise its submission to your journal for publication.

## Conflicts of Interest

The authors declare no conflicts of interest.

## Supporting information


**Figure S1:** Gene co‐expression networks of the purple, pink, magenta, and turquoise modules.
**Figure S2:** Gene co‐expression networks of the black, brown, and red modules.

## Data Availability

The data that support the findings of this study are available in GEO at https://www.ncbi.nlm.nih.gov/geo/, reference number GSE230809, GSE186542. These data were derived from the following resources available in the public domain: Original Dataset (GSE230809), https://www.ncbi.nlm.nih.gov/geo/query/acc.cgi?acc=GSE230809, Original Dataset (GSE186542), https://www.ncbi.nlm.nih.gov/geo/query/acc.cgi?acc=GSE186542.
