# Supplementary material for: Metabolic Dysregulation of FC3 Fibrochondrocytes via MDH2 Promotes Intervertebral Disc Degeneration
Source: J Cell Mol Med. 2026 Jul 2;30(13):e71246. doi: 10.1111/jcmm.71246 (PMC13329120; doi:10.1111/jcmm.71246)
Supplement: Supplementary file 1 — Figure S1: Gene co‐expression networks of the purple, pink, magenta, and turquoise modules. Figure S2: Gene co‐expression networks of the black, brown, and red modules. [file JCMM-30-e71246-s001.docx]

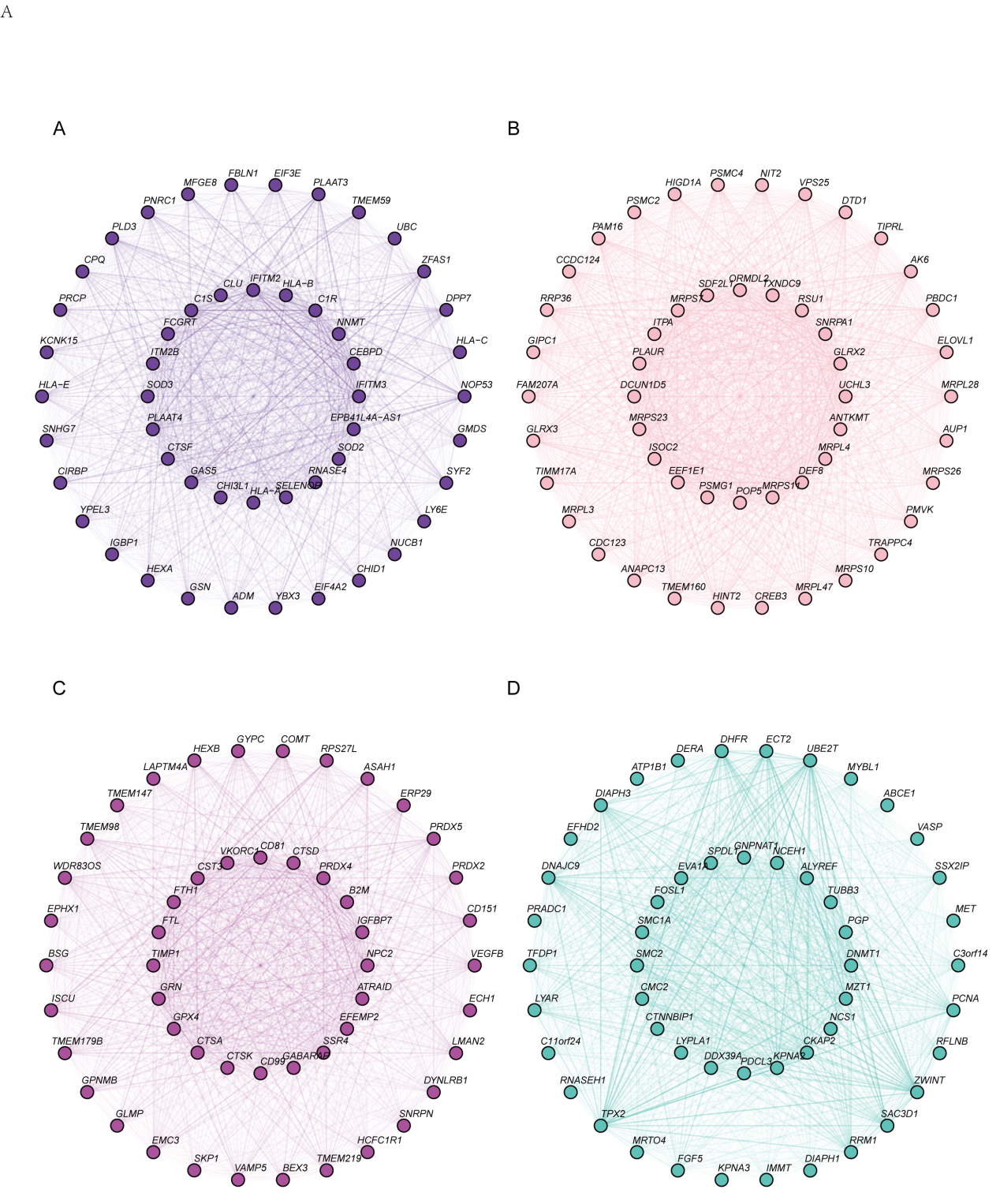


Supplementary Figure 1: Gene co-expression networks of the purple, pink, magenta, and turquoise modules

A: Visualization of the gene co-expression network for a key module (e.g., purple).B: Visualization of the gene co-expression network for a key module (e.g., pink).C: Visualization of the gene co-expression network for a key module (e.g., magenta).D: Visualization of the gene co-expression network for a key module (e.g., turquoise).


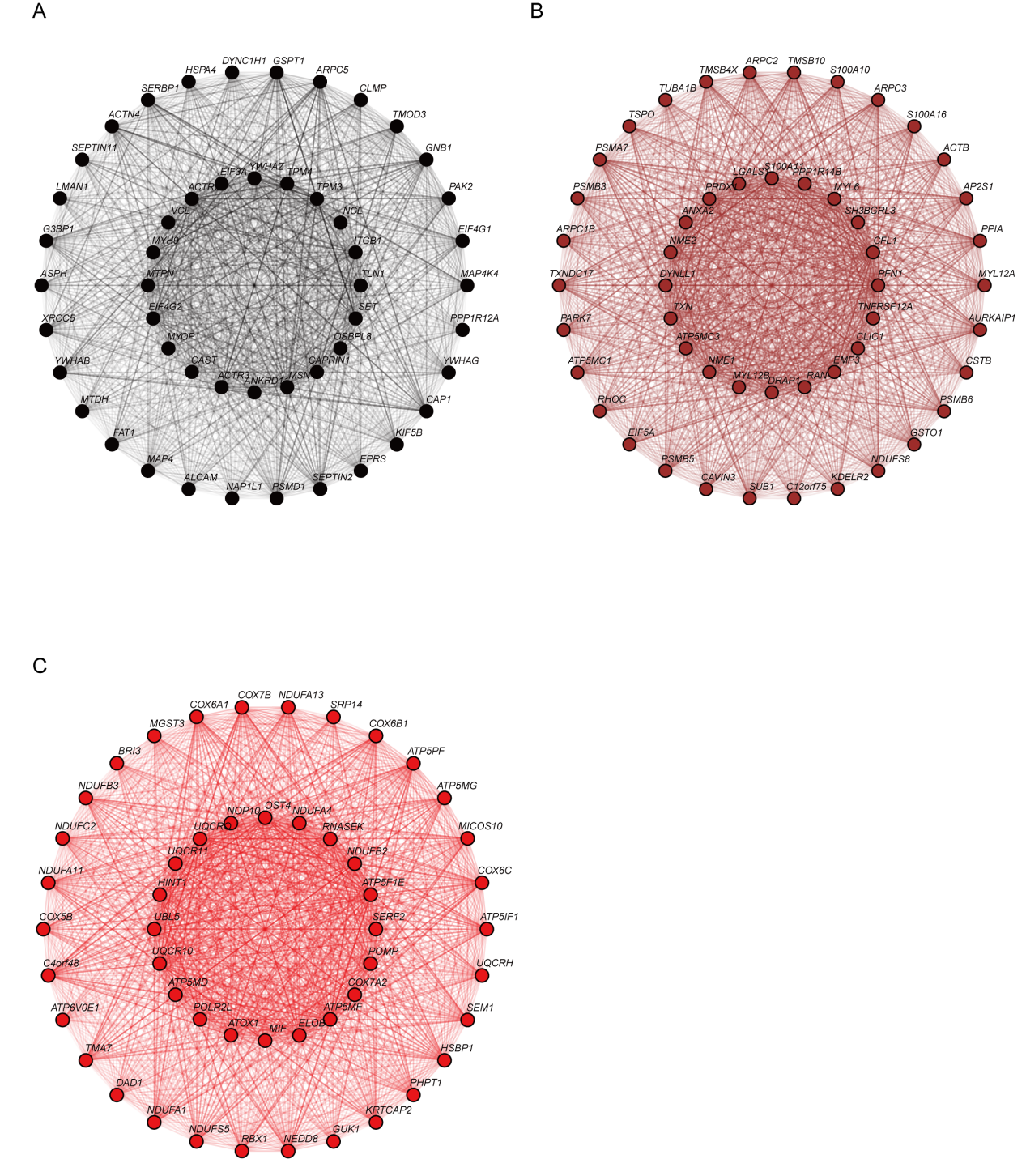


Supplementary Figure 2: Gene co-expression networks of the black, brown, and red modules

A: Visualization of the gene co-expression network for a key module (e.g., black).B: Visualization of the gene co-expression network for a key module (e.g., brown).C: Visualization of the gene co-expression network for a key module (e.g., red).
